# Lung cancer revealed by multiple metastases of the scalp

**DOI:** 10.11604/pamj.2016.24.290.10205

**Published:** 2016-07-29

**Authors:** Mohamed Fetohi, Tarik Namad

**Affiliations:** 1 Medical Oncology Department, Military Hospital Moulay Ismaïl, Meknès, Morocco

**Keywords:** Multiples skin metastases, scalp, lung cancer

## Image in medicine

Skin metastases of lung cancer are rare. They are symptoms of progressive disease and usually a sign of a poor prognosis. We report a case of 69-years-old man with no significant medical history, never smoker, which consulted a dermatologist for scalp nodules that appeared for more than 16 months in the scalp and gradually and slowly increased in size which the largest measure at admission between 1.5 cm and 4.0 cm. A biopsy was performed by the dermatologist and showed a scapular location of a squamous cell carcinoma. A brain CT objectified lesion of the vault of the skull next to a scalp injury and thoraco-abdominal pelvic CT showed a 3cm lesion in the lower lobe of the left lung with hilar lymphadenopathy ipsilateral. Bone scintigraphy showed secondary lesions in the thoracic spine (D6, D7) and lumbar spine (L2, L3) clinically asymptomatic. The patient is currently in 1st line chemotherapy (carboplatin-gemcitabine + Bisphosphonates) with a bad tolerance and poor response after the third cycle.

**Figure 1:**
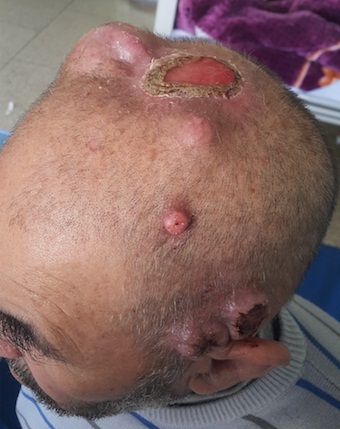
Lung cancer revealed by multiple metastases of the scalp

